# Female infertility and herbal medicine: An overview of the new findings

**DOI:** 10.1002/fsn3.2523

**Published:** 2021-08-15

**Authors:** Mohsen Akbaribazm, Nader Goodarzi, Mohsen Rahimi

**Affiliations:** ^1^ Fertility and Infertility Research Center Health Technology Institute Kermanshah University of Medical Sciences Kermanshah Iran; ^2^ Department of Basic Sciences and Pathobiology Faculty of Veterinary Medicine Razi University Kermanshah Iran; ^3^ Department of Parasitology and Mycology School of Medicine Student Research Committee Shahid Beheshti University of Medical Sciences Tehran Iran

**Keywords:** endometriosis, female infertility, isoflavonoid, medicinal plants, menopause

## Abstract

Infertility is defined as the failure to achieve a successful pregnancy after 12 months’ sexual activity that affects 15%–17% of couples in the world and about 50% of them are related to female infertility factors. In this study, using the PRISMA checklist and MeSH keywords, 128 articles were extracted from various databases (PubMed, Cochrane library, WHO, Iranmedex, Science Direct, SID, and Google Scholar search engine) without language and time restrictions, and 128 articles were selected after eliminating duplicate studies. In this review, we present some solid evidence for role of herbal medicine in the treatment of female infertility. The results of this study showed that different parts of some plants are rich in polyphenolic compounds (isoflavones and flavonoids) and other compounds which are beneficial to in reproductive health in women. The compounds in these plants, along with regulating the female endocrine pathways, and improving symptoms of menopause, treat female reproductive disorders such as polycystic ovary syndrome (PCOS), premature ovarian failure (POF), endometriosis, hyperprolactinemia, and hypothalamic dysfunction; moreover, because of their anticancer, antioxidant, and antidepressant properties, they can be used in traditional medicine or in the pharmaceutical industry as safe compounds in women's health.

## Graphical abstract

Herbs originate from plants and plant extracts from a wide variety of natural resources, including plant leaves, bark, flowers, roots, and fruits and berries. Evidence‐based herbal medicine may present an effective treatment for female infertility. In this review, we present some solid evidence for role of herbal medicine in the treatment of female infertility.

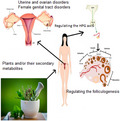

**Highlights**

1. The present review highlights some medicinal plants used in the treatment of woman disorders related to infertility.
2. Some plants are constituted of biological actives substances which have been used to treat reproductive dysfunction.
3. Some plants and/or their secondary metabolites regulated folliculogenesis and steroidogenesis.
4. Some plants and/or their secondary metabolites treat the female reproductive disorders such as polycystic ovary syndrome (PCOS), premature ovarian failure (POF), endometriosis, hyperprolactinemia, and hypothalamic dysfunction.

## 1 INTRODUCTION

One in six couples worldwide suffers from infertility, defined as failure to achieve successful pregnancy after one year of unprotected sex. About 50% of the reasons for infertility in couples are related to female disorders (Vander Borght & Wyns, 2018). Female infertility may be caused by an underlying variety of disorders, such as ovulation disorders, damages the fallopian tubes (tubal infertility), cervical disorders (benign polyps or tumors and cervical stenosis), and hormonal imbalances. These hormonal conditions include polycystic ovary syndrome (PCOS), endometriosis, premature ovarian failure (POF), hypothalamic dysfunction, hyperprolactinemia (too much prolactin), uterine fibroids, and pelvic inflammatory disease (PID) (Mustafa et al., 2019). The most important risk factors are smoking, heavy use of alcohol, chemotherapy or radiation therapy, long‐term use of high‐dosage nonsteroidal anti‐inflammatory drugs (NSAIDs), antipsychotic medications, consumption of recreational drugs such as marijuana and cocaine, obesity, increasing age, and sexually transmitted infections (STIs). Consequences of infertility in women are classified into two general categories.

The first category is related to physical disorders caused by infertility, and the second category includes psychosocial disorders. The physical symptoms of this disease include menstrual disorders (no periods, irregular periods, abnormal periods, painful periods, skin changes, changes in sex drive and desire, excessive hair growth (dark hair growth on the lips, chest, and chin) and weight gain. Psychosocial disorders caused by this disease include inter personal relationships problems, decreased self‐esteem, feelings of shame, social isolation, risk of harm to mental health, depression, anxiety, despair, guilt, and worthlessness (Abrao et al., 2013). Fortunately, today, it is possible for women to have children via various new therapies such as use of drugs for induction of ovulation (such as clomiphene and gonadotrophins), assisted reproductive technologies (ART) [such as in vitro fertilization (IVF) and intrauterine insemination (IUI)], egg and sperm donation, induction of ovulation and micronutrients have made it possible for infertile women to have children (Mascarenhas et al., 2017).

Various studies have shown the role of micronutrients in the treatment of female infertility alone and in combination with other treatments. These micronutrients include antioxidants, B vitamins, vitamin D, and fatty acids [saturated fatty acids, monounsaturated fatty acids (MUFS), polyunsaturated fatty acids (PUFAs), docosapentaenoic acid, eicosapentaenoic acid, linoleic acid, omega‐3, and omega‐6] (Silva et al., 2019; Naseri et al., 2019). Due to the negative effects of chemical drugs on reproductive health, high costs of drugs, and modern fertility treatment procedures, the tendency to use herbal medicines is increasing among women. Herbal medicine considered as suitable alternative to chemical medicines because of it the presence of various compounds with phytoestrogenic, antioxidant, and nutritional effects. Using estrogen‐mimetic phytoestrogens is one of the beneficial and healthy strategies to reduce the symptoms of menopause in women due to estrogen deficiency (Ascenzi et al., 2006). The aim of this study is to investigate the effects of different plants on female infertility.

## 2 METHODS (DATA EXTRACTION PROCESS)

**FIGURE 1:**
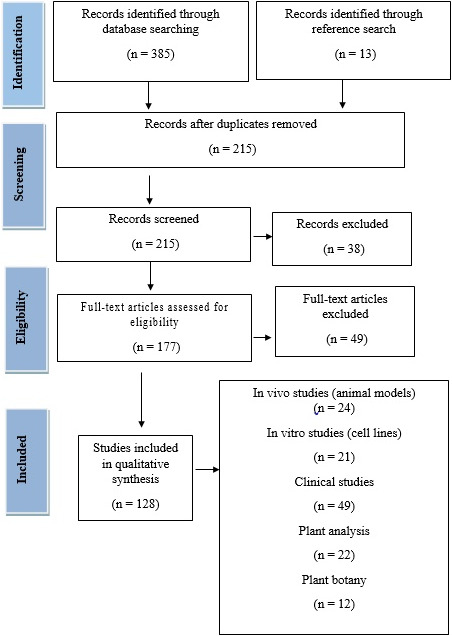
PRISMA checklist. Flowchart of the entrance steps of the article information

Overall, 128 related articles were selected for this review (PRISMA flowchart) (Figure 1). The articles were collected from searches in PubMed, Cochran library, WHO, Iranmedex, Science Direct, SID, and Google Scholar search engine for different types of plants, which have effect on fertility process using their scientific names. Then, we chose the plants that have positive effect on female fertility and searched articles with different keywords such as "the effect of (name of plant) on the female reproductive system or reproductive function or oogenesis." Original papers without language and time restrictions were chosen. 128 articles were selected after eliminating duplicate studies. Information from selected articles was classified according to the target effect of a plant extract on in vitro and in vivo (rodent and human female reproductive) function.

## 3 RESULTS & DISCUSSION

### 3.1 Plants and fertility

**FIGURE 2:**
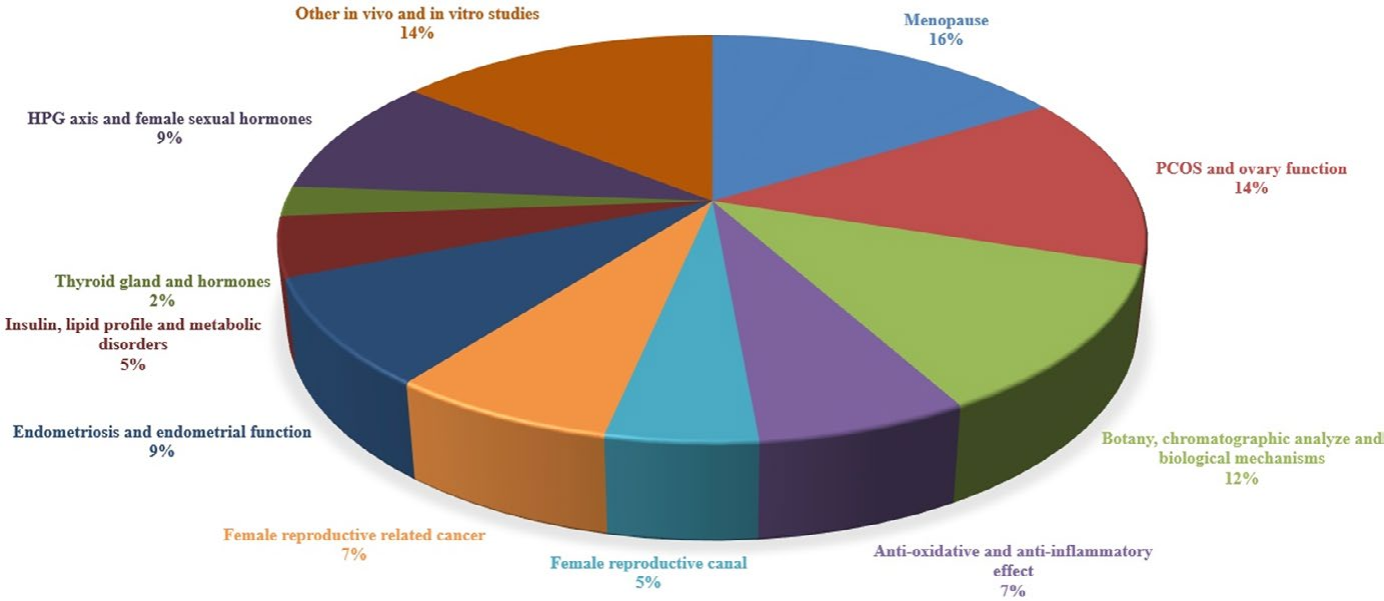
Distribution of papers based on subject

**FIGURE 3:**
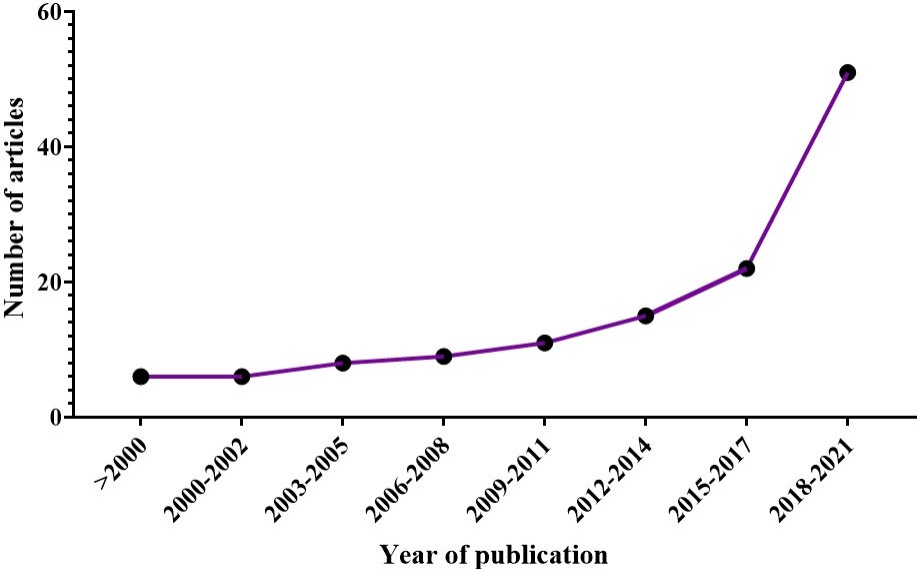
Distribution of papers by publication year

Most studies were "clinical human" (38%) and then "animal" studies (19%), and also based on the subject of the papers (Figure 1), most papers on the effects of different plants focused on "menopause" (16%) and "PCOS and ovarian function” (14%) (Figure 2). Also, based on time of publication of papers, most of the selected studies (48%) were published in the period 2018–2021 and the upward slope of studies has intensified from 2014 to 2021 (Figure 3).

Medicinal plants studied in article are able to increase fertility in a variety of ways, including amplifying the hypothalamic–pituitary–gonadal axis (HPG axis) and interaction with estrogen receptors (α and β). Also they (plants) can prevent the reproductive transmitted bacterial/viral/fungal infections and inflammatory reactions/hypersensitivity/autoimmune disorders and, finally, providing proper nutritional conditions to regulate ovulation, implantation, uterine embryo tolerance, and fetal maturity (Akbari Bazm et al., 2019; Jiang et al., 2019).

Two estrogen receptors α/β (ERα/ERβ) have been identified to date, and the physiological responses to estrogen are known to be mediated within specific tissues by at least these two receptors. Estrogen receptors α (ERα) are expressed in breast and uterine, while ovarian tissues and estrogen receptors β (ERβ) are expressed in bone and blood vessels. The ERs are classified as nuclear hormone receptors and act as ligand‐activated nuclear transcription factors (Paterni et al., 2014).

Many plants produce compounds that possess estrogenic activity (called phytoestrogens) in animals. These compounds (such as formononetin, genistein, daidzein, and biochanin A) are most likely to have higher affinity to the estrogen receptor β (Akbaribazm et al., 2020a; Ye & Shaw, 2020). Although these derivatives bind more strongly to ER β than α, the concentration required for the induction is almost the same in the case of ER β and ER α and is much higher than what is expected from the binding affinity (Barnes et al., 2000).

These compounds reduce bone loss due to menopause, and therefore, the level of uric acid deoxypyridinol decreases, but osteogenic indices such as alkaline phosphatase and osteocalcin remain unchanged. Also, phytoestrogens are used to treat menopausal symptoms (such as reduce hot flashes, vaginismus, and dyspareunia) because these compounds have vasoconstrictive properties (Cheng et al., 2007). Also, compounds in medicinal plants such as flavonoids and isoflavones can lower blood sugar, improve lipid profile by lowering triglycerides and LDL, and increase HDL, which are important in reproductive cycles (Akbaribazm et al., 2021; Han et al., 2002).

In addition, various studies have shown that plants containing polyphenolic compounds suppress the growth of breast tumors by inhibiting pathways such as insulin‐like growth factor 1 (IGF‐1)/P13K/Akt and ERK1/2 MAPK‐Bax (Park et al., 2018, Akbaribazm et al., 2020b). Studies on the effects of plant polyphenols (such as isoflavones) on the skin show that plant extracts containing these compounds increase the synthesis of hydroxyproline as an indicator of collagen and elastic fibers synthesis, vasodilatation (increased VEGF levels) of skin vessels, regulate sweat and sebaceous gland secretion, and regulate collagen synthesis in the growth of hair layers from the effects of menopause and infertility symptoms (Izumi et al., 2007).

### 3.2 *Punica* *granatum* (Pomegranate)

*Punica granatum* (pomegranate) has spread to most parts of the world, including Asia, the Middle East, and the Mediterranean countries (Melgarejo et al., 2020). Pomegranate contains a large percentage of water and is rich in vitamin C and polyphenols such as anthocyanins, punicalagin, ellagic, and gallic acids. Pomegranate seeds contain phytoestrogens likes genistein, daidzein, coumestrol, glutamic amino acids, and aspartic acids (Battineni et al., 2017). An animal study on rats with PCOS shows that pomegranate extract due to the presence of phytoestrogens can regulate and reduce PCOS symptoms. The extract of this plant increases mucus secretion by increasing uterine blood flow (vasodilatation) and also increases the thickness of the uterine wall. This increase in mucosal secretions through anti‐inflammatory mechanisms enhance the implantation rate (Hossein et al., 2015; Promprom et al., 2010).

Pomegranate peel contains calcium and tannins, which Mohammadzadeh et al. (2019) in a triple‐blind randomized controlled clinical trial on 110 normal women showed increase of sexual satisfaction of women after using them as a gel, and also reduction of inflammatory and infectious symptoms in their reproductive canal. Study of different cell lines of human breast (MCF‐7, MDA MB‐231) endometrial (HEC‐1A), cervical (SiHa, HeLa), ovarian (SKOV3) carcinoma, and normal breast fibroblast (MCF‐10A) cells showed that pomegranate extract estrogen receptor modulators (SERMs) by binding to ERs inhibit the growth of these cell lines in vitro and in vivo (in ovariectomized mice) models and prevent the proliferation of these cells (Sreeja et al., 2012).

In a randomized controlled triple‐blind parallel trial study on 23 women with PCOS, it was found that pomegranate fruit extract improved the serum levels of sex hormones (testosterone reduction) and their lipid profile (Esmaeilinezhad et al., 2019). A study on PCOS‐induced rats, pomegranate fruit extract was found to increase serum estrogen levels and reduce symptoms after 81 days (Hossein et al., 2015).

### 3.3 *Matricaria* *chamomilla* (Chamomile)

*Matricaria chamomilla* (Chamomile) is a plant of the chicory family and contains flavonoid compounds and antioxidants such as gallic acid, camazelin, farnesene, matricin, coumarin derivatives, apigenin, and choline (Avallone et al., 2000). In a study on the growth and maturation of isolated mouse ovarian follicles in a three‐dimensional culture system, it was shown that chamomile extract increased progesterone, 17β‐estradiol, and dehydroepiandrosterone levels in the culture medium, decreased ROS, follicular diameter, and antrum formation, and prolonged the survival of oocytes (Shoorei et al., 2018). It has been shown that in gonadectomized mice, chamomile extract improves estrogen‐dependent sexual parameters including hair growth, temperature changes, and the menstrual cycle (Kesmati et al., 2006). Also, in a double‐blinded clinical trial by Gholami et al. (2016) on 80 post‐term pregnant women with a gestational age of 40 weeks or more, it was found that after one week of taking chamomile extract‐containing capsules, labor symptoms began to appear, and compared to the control group, labor pain and contraction duration decreased (Gholami et al., 2016).

The phytoestrogenic compounds of chamomile cause galactogogue effects by acting on dopamine receptors, and in human studies, the extract of this plant has been shown to increase lactogenesis in lactating woman (Silva et al., 2018). In a pilot randomized controlled trial on 56 women with idiopathic hyperprolactinemia, the women treated with chamomile syrup (5 ml, twice daily) showed a decrease in prolactin levels after four weeks compared with the placebo group (Kabiri et al., 2019), suggesting a role for chamomile in the modulation of prolactin secretion in women by acting on dopamine receptors. In another randomized clinical trial on 130 women, chemocline odor improved labor contractions during delivery (Heidari‐Fard et al., 2018). Chamomile extract has been suggested to prevent postpartum hemorrhage and alleviate pain in women by inhibiting COX‐2, representing even better effects compared to chemical drugs such as mefenamic acid and NSAIDs (Abedian et al., 2016).

### 3.4 *Vitex* *Agnus‐castus* (Verbenaceae)

Traditionally, *Vitex agnus castus* (a species of the Verbenaceae family) has been used for alleviating the menstrual problems resulting from corpus luteum deficiency, including spasmodic dysmenorrhea and premenstrual symptoms, certain menopausal conditions, and insufficient lactation, as well as for treating acne (Zamani et al., 2012). The LC‐ESI/MS analysis of Verbenaceae plants’ extracts confirmed the presence of compounds such as orientin, casticin, rutin, rosmarinic acid glycoside, quercetagetin trimethyl ether, biochanin A, genistein, syringetin C glycoside, agnuside, kaempferol‐7‐O glucuronide, luteolin‐7‐O‐glucoside, homorientin, and isovitexin. Most of these compounds are isoflavones and flavonoids that have a high affinity for estrogen receptors (ERs) and are beneficial to improve women's sexual health (Mari et al., 2015). The flavonoids of this plant by increasing the release of nitric oxide (NO) and cyclic guanosine monophosphate (cGMP) from vascular endothelium increase endometrial blood flow, and its isoflavones reduce the release of the prolactin and FSH hormones by affecting the HPG axis (Amégbor et al., 2012; Goodarzi & Akbari, 2016).

Human studies have shown the effectiveness of the plant species of the Verbenaceae family in the treatment of gynecological diseases and premenstrual symptoms such as depression, sadness, and irritability (Abdnejad & Simbar, 2016), as well as irregular menstrual bleeding, hyperprolactinemia, dysmenorrhea, and menopausal problems (Sadeghi et al., 2019). By acting on dopamine receptors, Verbenaceae extract reduces prolactin secretion, modulates the release of FSH and LH, increases serum levels of estrogen and progesterone, and ultimately improves sexual function (Ibrahim et al., 2008).

The extract of this plant also stimulates the secretion of corpus luteum after ovulation to produce progesterone, which ultimately regulates female sexual cycle (Askari, 2017). In an animal study by Yakubu and Akanji (2011), it was observed that the serum levels of estrogen and progesterone increased in the group receiving Verbenaceae extract compared to the control group while LH and prolactin, as sexual function disruptive hormones, decreased (Yakubu & Akanji, 2011). Also, a study in rats with induced PCOS showed that the extract of this plant reduced the number of preantral and antral follicles and corpus luteum in comparison with the control group after 28 days. Also, the diameter of antral follicles and the thicknesses of follicular theca and ovarian tunica albuginea increased compared to the control group (Jelodar & Karami, 2013).

### 3.5 *Withania* *somnifera* (Ashwagandha)

*Withania somnifera* (Ashwagandha), also called Indian ginseng, belongs to the Solanaceae family, showing beneficial effects in women with problems in conceiving. This wild plant grows in dry and hot‐semiarid climate, such as in the southern Mediterranean region, Canary Islands, and northern Africa to northern India (Iran, Jordan, Sudan, Palestine, Afghanistan, and Egypt) (Peters, 2018). In traditional medicine, the plant has been recommended for the management of premature ejaculation, polyarthritis, painful swellings, lumbago, oligospermia, vitiligo, general debility, ulcers, impotency, uterine infections, leucorrhoea, and orchitis (Ali et al., 2017). The LC‐ESI/MS analysis of Ashwagandha extract confirmed the presence of compounds such as anaferin, anahygrine, hygrine, cuscohygrine tropine, pseudotropine, withananine, pseudowithanine, somnin, and somniferine‐3‐tropyltigloate (Nasimi et al., 2018). Most of the plant's compounds are polyphenols (isoflavones and flavonoids) that can play an estrogenic role.

In a study by Saiyed et al. (2016) on letrozole‐induced polycystic ovarian syndrome in rats, it was found that the serum level of LH decreased; FSH level increased, and preantral and antral follicles and corpus luteum reduced in comparison with the control group in 22 days (Saiyed et al., 2016). In the study of Bhattarai et al. (2010), it was found that Ashwagandha extract via GABA mimetic properties increased the secretion of gonadotropin hormones and finally improved oogenesis, which was proposed to be due to boosting the HPG axis and improving serum estrogen balance (Bhattarai et al., 2010). Various studies have shown the anticancer properties of the plant extract against different breast tumor cell lines (MCF‐7 and MDA‐MB‐231) in vitro, as well as in vivo (4T1 tumor‐bearing Balb/c mice) via reducing the growth of tumor cells. Ashwagandha extract has also been shown to improve the survival and life quality of patients with breast cancer (Bazm et al., 2018; Biswal et al., 2013).

### 3.6 *Trifolium* *pratense* (Red clover)

The clover (Trifolium) family is cultivated all over the world, and its members are used in the food, pharmaceutical, and cosmetics industries. Also, different parts of this plant contain essential minerals (P, Mg, Cr, K, Ca, Na, and Fe) that, along with other compounds, can regulate many molecular pathways and biological processes, especially the immune system (McKenna et al., 2018). The LC‐ESI/MS analysis of red clover extract confirmed the presence of compounds such as cinnamic acid, 3‐O‐(Z)‐pcoumaroylquinic acid, epigallocatechin, caffeic acid, quercetin‐3,7‐diglucoside, kaempferol‐3,7‐di‐O‐glucoside, myricetin‐3‐Orhamnoside, fraxidin, vitexin‐O‐(maloyl) rhamnoside, formononetin, genistein, biochanin A‐7‐glucoside, daidzein 7‐O‐D‐glucoside, and apigenin‐7‐O‐glucoside (Akbaribazm et al., 2020c). The phytoestrogenic compounds of red clover simulate the production of hormones in the female body and decrease the severity and frequency of menopausal symptoms by binding to ERβ (Hidalgo et al., 2005; Salimpour et al., 2007).

In a randomized and placebo‐controlled trial, 190 postmenopausal women were treated with red clover extract (28.6 mg kg^‐1^ day^‐1^) over 90 days, which insignificantly reduced the symptoms of depression and anxiety and improved hair loss and skin characteristics in these women compared to the placebo group (Tice et al., 2003). In a prospective, randomized, double‐blinded, placebo‐controlled trial by Del Giorno et al. (2010), 40 mg/kg of red clover over a 12‐month period did not significantly improve menopausal symptoms and sexual desire in 120 women with menopausal symptoms aged 45–65 years (Del Giorno et al., 2010). In another randomized, double‐blinded, placebo‐controlled trial, hormonal levels and endometrial thickness improved, and hot flushes reduced by 44% in 30 postmenopausal women treated with 80 mg/kg red clover extract for more than 12 months as compared with the placebo group (van de Weijer & Barentsen, 2002).

Red clover extract (375 and 750 μg/g) after 21 days increased the weight of the uterus (via binding to Erα), and through binding to ERβ, increased mammary gland development and plasma prolactin level, and decreased bone loss in ovariectomized rats (Santell et al., 1997). The doses of 200 and 400 mg/kg of red clover hydroalcoholic extract suppressed the proliferation of triple‐negative breast tumor cells and their metastasis to the lung and brain in 4T1 tumor‐bearing Balb/c mice after 35 days, partly via activating the ERα signaling pathway, decreasing 17 β‐estradiol levels, and modulating apoptosis‐related pathways (Akbaribazm et al., 2020b; Akbaribazm et al., 2020a).

In an in vitro study, the endometrial glandular cells were isolated from five premenopausal and nonpregnant women whose endometrium was in the proliferative phase, and the cells were incubated with red clover extract, which significantly decreased ER‐α and increased ER‐β mRNA expressions and also suppressed the secretion of cytokines such as TNF‐α and IL‐1α. These results suggested that the expression of ER‐α/β in endometrial glandular cells might be regulated by phytoestrogens at the mRNA and protein levels (Staar et al., 2005). In addition, in a study by Lian et al. on animal models (2001), a single subcutaneous administration of genistein (1 mg/30 g body weight) in short‐term (2 weeks) and daidzein (1 mg/30 g body weight) in long‐term (30 weeks) inhibited the expression of estrogen‐dependent genes (c‐fos and c‐jun) and suppressed IL‐lα and TNF‐α cytokines through cytokine‐ and estrogen receptor‐mediated pathways, which are important in the proliferation and differentiation of endometrial cells (Lian et al., 2001).

### 3.7 *Camellia* *sinensis* (Chinese tea)

*Camellia sinensis* (Chinese tea) belongs to the Theaceae family and grows in East Asia, the Indian Subcontinent, and Southeast Asia, but it is today cultivated across the world in tropical and subtropical regions (Mahmood et al., 2010). The LC‐ESI/MS analysis of *C. sinensis* extract confirmed the presence of compounds such as galloylquinic acid, epigallocatechin, epicatechin, succinic acid, gallocatechin, strictinin, apigenin glucosyl arabinoside, quercetin, myricetin, genistein, biochanin A‐7‐glucoside, daidzein 7‐O‐b‐D‐glucoside, apigenin‐7‐O‐glucoside, cyanidin, delphinidin glycoside, kaempferol, p‐coumaroyl glucosyl, rhamnosyl galactoside, malic acid, and pyroglutamic acid (Jeszka‐Skowron et al., 2018; Ku et al., 2010).

As expected, due to the fact that most of the plant's constituents are isoflavones and flavonoids, it shows a variety of therapeutic properties. The compounds in this plant have antioxidant and phytoestrogenic properties and restored the secretion and concentration of sex hormones including LH, FSH, estradiol, and testosterone in female Wistar rats with letrozole‐induced PCOS (Khodarahmi et al., 2020). Taking into the mind that the aromatase enzyme plays an important role in the synthesis of estradiol in ovarian granulosa cells, the isoflavones of this plant were shown to inhibit the production of this enzyme by granulosa cells and therefore reduce estradiol production in a dose‐dependent manner (Morshedi et al., 2016).

In a study on bilaterally oophorectomized rats, Das et al. (2005) showed that *C. sinensis* extract in a dose‐dependent manner (2.5%, 1 ml/100 g body weight/day for 28 days) reduced estrogen‐dependent menopausal symptoms and prevented osteoporosis by increasing bone mineral reserves (Das et al., 2005). Another study showed that the extract of this plant in a dose‐dependent manner (100, 200, and 400 mg/kg for 15 days) increased the maturity and secretory activity of mammary sinuses by regulating prolactin secretion (Al‐Snafi et al., 2015). Also, Ratnasooriya and Fernando (2009) investigated pregnancy outcomes in the rats exposed to *C. sinensis* extract during early (days 1–7), mid (days 8–14), and late (days 15–21) pregnancy period and found improvements in the implantation index (number of viable implants, pre‐implantation loss, and postimplantation loss), gestation index, and gestation length compared with the control group (Ratnasooriya and Fernando, 2009).

### 3.8 *Phoenix* *Dactylifera* (Date palm)

*Phoenix Dactylifera* (date palm) belongs to the palm (Arecaceae) family, and its fruit has been used in Iranian, ancient Rome, ancient Egyptian, Chinese, and Greek traditional medicine to increase sexual potency and pleasure in men and women and reduce menopausal symptoms in women (Qadir et al., 2020). This plant contains essential elements such as cobalt, copper, fluorine, magnesium, manganese, selenium, and zinc and also vitamins (A, B1, B2, B3, B5, B6, B9, C, E, and K) and shows beneficial effects in improving female fertility. Various studies have shown the anticancer and anti‐inflammatory effects of this plant, its nephroprotective and hepatoprotective effects, as well as its protective effects against toxicity‐induced reproductive dysfunction in female/male animal models (Taleb et al., 2016). The LC‐ESI/MS analysis of the fruit and seed of this plant showed the presence of polyphenolic compounds (isoflavonoids and flavonoids) such as gallic acid, biochanin A‐7‐glucoside, daidzein 7‐O‐D‐glucoside, syringic acid, protocatechuic acid, protocatechuic acid, genistein, p‐hydroxybenzoic acid, proantosinidin, vanillic acid, caffeic acid, syringic acid, p‐coumaric, ferulic acid, mcoumaric acid, and o‐coumaric acid (Souli et al., 2018). Studies show that saponins in this plant enhance the blood flow toward the female reproductive system by releasing NO and stimulating the HPG axis (Bahmanpour et al., 2006).

A study in mice showed that *Phoenix dactylifera* extract due to the presence of gonadotropin‐like biocomponents such as rutin, sterols, carotenoids, and androsterone stimulates oogenesis and increases the number of antral and secondary follicles and the growth and maturation of preantral follicles. In PCOS, an elevated LH to FSH ratio increases the synthesis of androgens, insulin, and insulin‐like growth factors (IGFs), which in turn increases the number of cystic follicles (Hosseini et al., 2014; Salek Abdollahi et al., 2015). However, the study of Karimi Jashni et al. (2016) showed that the consumption of *P. dactylifera* extract due to the presence of estrogen‐like compounds reduced the LH to FSH ratio and the number of cystic follicles, modulated estrogen and androgen synthesis, and increased the number of secondary and antral follicles (Karimi Jashni et al., 2016). The estrogenic compounds of this plant extract can be used to treat uterine disorders as they have been shown to reduce endometrial tissue degeneration, necrotic patches, and hyperplasia in endometrial glands (El‐Mansi et al., 2019).

### 3.9 Cinnamomum

From 250 genera of Cinnamomum around the world, 33 present therapeutic and nutritional effects. The four species of *C. verum* (true cinnamon, Sri Lankan, or Ceylon cinnamon), *C. burmannii* (Java or Indonesian cinnamon), *C. cassia* (Chinese cinnamon), and *C. loureiroi* (Vietnamese or Saigon cinnamon) are important herbs used for their therapeutic properties in traditional medicine and the pharmaceutical industry (Kumar et al., 2019). Cinnamomum extracts and their effective compounds have also been used for treating asthma, bronchitis, diarrhea, headache, inflammation, cardiac disorders, and PCOS, as well as to increase female/male sexual potency and female sexual desire (Zaidi et al., 2015). The major constituents of this plant are polyphenols (flavonoids and isoflavones) such as eugenol, pyrogallol, cinnamic acid, ferulic acid, caffeic acid, gallic acid, protocatechuic acid, oleic acid, p‐hydroxybenzoic acid, quercetin, epigallocatechin, daidzein 7‐O‐D‐glucoside, epicatechin, succinic acid, biochanin A‐7‐glucosid, gallocatechin, strictinin, apigenin glucosyl arabinoside, quercetin, myricetin, genistein, and apigenin‐7‐O‐glucoside (Singh et al., 2021).

*Cinnamomum cassia* and *C. verum* extracts were shown to inhibit spontaneous and oxytocin‐induced uterine contractions in animal models in a dose‐dependent manner through suppressing uterine intracellular Ca^2+^ influx, cyclooxygenase‐2 (COX‐2) activity, and prostaglandin F_2α_ (PGF_2α_)‐dependent pathways (Sun et al., 2016). Women with primary dysmenorrhea suffer from menstruation‐associated symptoms such as prolonged bleeding form myometrium blood vessels and severe uterine contraction and pain due to endometrial ischemia caused by the release of uterine PGF_2α_ and COX‐2 (Pan et al., 2014). PGF_2α_ and COX‐2 activate myosin light‐chain kinase (MLCK) by releasing Ca^2+^ and forming the Ca^2+^‐calmodulin complex, leading to severe uterine contraction and pain (Arrowsmith & Wray, 2014). Although NSAIDs, such as ibuprofen, as nonspecific inhibitors of the COX‐1 and COX‐2 enzymes, reduce the painful symptoms of primary dysmenorrhea, their usage is limited due to the incidence of NSAID‐related disorders, including female infertility, renal failure, hepatotoxicity, and myocardial toxicity (Zahradnik et al., 2010).

Meanwhile, polyphenolic compounds in Cinnamomum alleviate dysmenorrhea pain and bleeding by suppressing L‐type Ca^2+^ channels and inhibiting Ca^2+^ release, as well as inhibiting the enzymes involved in arachidonic acid metabolic pathways, COX enzymes, and PGs‐related pathways without disrupting other organs in the body (Alotaibi, 2016). In addition, antifungal compounds in Cinnamomum effectively eradicated Candidiasis infections upon local application (gel and lotion containing the plant extract) in the vagina and systemic administration, which can prevent fungal‐induced infertility (Essid et al., 2017). Endometriosis is a clinical disease resulting from hypoestrogenism or pelvic surgeries, leading to ovaries’ poor function and quality (Kudoh et al., 1997). Today, the most common treatment for this condition is to block the secretion of estrogen from ovaries (Altintas et al., 2008). Due to the serious side effects of drugs such as aromatase inhibitors, gestrinone, and gonadotropin‐releasing hormone (GnRH) antagonists, the safest alternative to pharmaceutical treatments is to use the extracts of the plants containing polyphenols (isoflavonoids and flavonoids), like Cinnamomum, that can reduce estrogen secretion and prevent endometriosis progression via their antioxidant and anti‐inflammatory properties (Ji et al., 2011).

Studies show that insulin resistance plays a key role in the development of PCOS symptoms. Also, according to available evidence, hyperinsulinemia and insulin resistance not only cause metabolic problems, but also play an important role in the pathogenesis of fertility problems in women with PCOS (Khan et al., 2003). Cinnamon activates the glycogen synthase enzyme and inhibits the activity of glycogen synthase kinase 3 (GSK3), leading to a rise in glucose uptake. Cinnamon also induces the insulin receptor kinase enzyme and inhibits insulin receptor dephosphorylation. All of these effects lead to a decrease in insulin resistance (Rashidlamir et al., 2013). In a randomized controlled trial, Kort and Lobo (2014) showed that six months of treatment with Cinnamomum improved the menstrual cycle, insulin resistance, and androgen secretion in women with PCOS (Kort & Lobo, 2014). Studies also showed that Cinnamomum modulated the HPG axis and increased the secretion of gonadotropin hormones (GnRH) partly via inducing norepinephrine and NO production through the act of compounds like delta‐Cadinene (Parvizi & Ellendorff, 1982).

### 3.10 *Foeniculum* *Vulgare* (Fennel)

*Foeniculum Vulgare* (fennel) with yellow flowers and feather‐like leaves belongs to the Umbelliferae (Apiaceae) family and is native to the Mediterranean region, western Asia, and eastern Europe. It is a popular herb with a long history of usage as a traditional medicine (Grover et al., 2013). A series of studies have shown that fennel has beneficial effects against numerous infectious disorders of fungal, bacterial, mycobacterial, viral, and protozoal origin, as well as antitumor, antioxidant, cytoprotective, chemopreventive, hypoglycemic, hepatoprotective, and estrogenic properties (Badgujar et al., 2014). The major constituents of this plant are polyphenols (flavonoids and isoflavones) such as quercetin‐3‐glucuronide, isoquercitrin, quercetin‐3‐arabinoside, 3‐O‐caffeoylquinic acid, 4‐O‐caffeoylquinic acid, 5‐O‐caffeoylquinic acid, 1,3‐O‐di‐caffeoylquinic acid, 1,4‐O‐di‐caffeoylquinic acid, kaempferol‐3‐glucuronide, kaempferol‐3‐arabinoside, isorhamnetin glucoside, rosmarinic acid, chlorogenic acids, Quercetin‐3‐O‐galactoside, kaempferol‐3‐O‐rutinoside, kaempferol‐3‐O‐glucoside, isorhamnetin 3‐O‐rhamnoside, quercetin, and kaempferol (Faudale et al., 2008; Parejo et al., 2004).

Idiopathic hirsutism is characterized with changes in the levels of androgen and estrogen and abnormality in the ovulatory menstrual cycle. A double‐blinded placebo‐controlled trial by Javidnia et al. (2003) showed that a cream containing fennel extract improved hirsutism in a dose‐dependent manner (1% and 2% concentrations) compared to the placebo group, resulting in the mean hair diameters of 18.3%, 7.8%, and 0.5% in the 2% and 1% cream and placebo, respectively (Javidnia et al., 2003). Fennel contains effective compounds that can modulate steroidogenesis pathways, including anol (demethylated anethole), which acts similar to catecholamines and induces the secretion of prolactin. Diosgenin is another constituent of fennel, which is a sapogenin steroid compound and modulates DHEA synthesis (Sautour et al., 2004). Due to the presence of dianethole and photoanethole, fennel is known as a galactagogue plant that increases the growth of the mammary glands of the lobule‐alveolar system and induces prolactin secretion by competing with dopamine for binding to dopamine receptors (Rahimi & Ardekani, 2013).

A study on rats showed that low doses (50 µg/100 g body weight) of fennel extract after 10 days caused vaginal cornification and induced the estrus cycle while at moderate doses (250 µg/100 g body weight), and it increased the weight and volume of genital organs (mammary glands, oviduct, endometrium, myometrium, cervix, and vagina) in female rats (Mallni et al., 1985). Fennel extract reduced the frequency of uterine contractions by affecting the synthesis of oxytocin and PGF_2_ and alleviated dysmenorrhea pain at different doses. These effects were even more potent than those of mefenamic acid (Ostad et al., 2001). A study on ovariectomized rats showed that 500–1000 mg/kg doses of fennel extract after 30 days increased mineral density and the synthesis of collagen fibers in bones in a dose‐dependent manner through alkaline phosphatase‐related pathways, suggesting a role for this plant in preventing osteoporosis, especially in postmenopausal women (Tanira et al., 1996).

### 3.11 *Nigella* *sativa* (Black seed‐ Ranunculaceae)

This plant belongs to the Ranunculaceae family and has a height of 30–60 cm and two to three pinnatisect leaves. The plant grows in many parts of the world, including Eastern Europe, West Asia, and Southeast Asia (Ijaz et al., 2017). Extracts of its various parts such as seeds, leaves, flowers, and stem are used to treat various diseases such as gastrointestinal disorders, headache and migraine, PCOS, male infertility, diabetes, renal injury, hyperlipidemia, stress and depression, neurological disorders, respiratory diseases, liver disorders, and cancers, as well as to mitigate menopausal symptoms (Akbari et al., 2017; Koshak et al., 2017; Majeed et al., 2020). Most of the active ingredients of this plant are polyphenols, especially flavonoids such as kaempferol 3‐glucosyl, quercetin 3‐(6‐feruloylglucosyl), apigenin, catechin, epicatechin, p‐coumaric acid, syringic acid, and chlorogenic acid (Ahmad et al., 2014; Saleh et al., 2018). Various studies have shown that *Nigella sativa* extract due to the presence of phytoestrogenic and flavonoid compounds reduces the number of ovary cysts induced by exposure to estradiol valerate, letrozole, and dehydroepiandrosterone in different animal models (rats and mice) of PCOS. The extract of this plant has been suggested to improve PCOS by upregulating the mRNA expression of epigenetic‐related (Dnmt1 and Hdac1) and maternally derived genes (Mapk and Cdk1), reducing ROS, and affecting the HPG axis (i.e., suppressing LH and estrogen secretion and also boosting FSH levels) (Khani et al., 2021).

Long‐term use of the extract of *N. sativa* due to the presence of phytoestrogens can reduce testosterone levels, exerting a negative feedback on LH. On the other hand, LH is probably produced to a lesser extent following a decrease in androgens, reducing the dominant effect of LH on FSH. In addition, *Nigella sativa* extract may reduce LH dominance over FSH by inhibiting nitric oxide and leptin‐releasing neurons that are directly involved in the synthesis of LH from the anterior pituitary gland, thereby increasing ovulation in women with PCOS (Eini et al., 2020). A study by Parhizkar et al. (2016) on the ovariectomized rats treated with *N. sativa* extract showed that the extract of this plant, in a dose‐dependent manner (300 to 1200 mg/kg), reduced menopausal symptoms including uterine weight/edema loss and decreased serum estradiol level and the number of inflammatory cells in the vagina compared with the control group (Parhizkar et al., 2016). The doses of 100 to 400 mg/kg of *N. sativa* extract decreased the serum level of estradiol and increased prolactin in a dose‐dependent manner in pregnant rats with hypothyroidism, which can be due to the stimulatory effects of phytoestrogens of this plant on dopaminergic neurons, reducing the risk of hypothyroidism (Pakdel et al., 2017).

### 3.12 *Glycyrrhiza* *glabra* (Licorice‐ Liquorice)

*Glycyrrhiza glabra* (Licorice‐ Liquorice) is an herbaceous perennial plant belonging to the Fabaceae family. The plant is about 50–100 cm high and has 7–12 pinnate leaves containing 9–17 leaflets with purple to pale whitish blue flowers (1‐cm‐long), as well as 3‐cm‐long pod fruits containing several seeds. This plant grows in most parts of the world but is native to western Asia and southern Europe (especially France, Uzbekistan, China, and Iran) (Öztürk et al., 2018). The plant contains a variety of phytoestrogens and issues potent antidiabetic, spasmolytic, antidepressive, laxative, antiulcer, and anti‐inflammatory effects (Duan et al., 2020). A dose higher than 2 mg kg^−1^ day^−1^ of pure glycyrrhizic acid (i.e., the main constituent of liquorice) may result in side effects such as hypokalemia, hypertension, apparent mineralocorticoid excess (AME) syndrome (via inducing sodium retention and suppressing the renin‐angiotensin‐aldosterone system), muscle weakness, and even death (Dastagir & Rizvi, 2016; Vergoten & Bailly, 2020). The LC‐ESI/MS analysis of licorice extract confirmed the presence of compounds such as matrine, oxymatrine, ferulic acid, mangiferin, glycyrrhizin, licuraside, licochalcon A/B, licoflavon, glycyrol, formonetin, neoisoliquiritin, isoviolanthin, shaftoside, glycyrrhisoflavon, glabron, licoflavonol, glycycumarin, glycyrrhetinic acid, liquiritigenin, isoliquiritigenin, and liquiritin (Montoro et al., 2011; Zhang et al., 2018). Due to high amounts of phytoestrogenic compounds, this plant can be beneficial in treating estrogen‐dependent diseases such as breast cancer, endometriosis, PCOS, and POF (Youseflu et al., 2020). Studies show that licorice inhibits two enzymes [3β‐hydroxysteroid dehydrogenase (3HSD) and 17‐hydroxysteroid dehydrogenase (17HSD)], stimulates the activity of aromatase, and also affects the activity of 5α‐ and 5β‐reductase enzymes, all of which are involved in the synthesis and metabolism of androgens and estrogens (Shimoyama et al., 2003). Due to the presence of phytoestrogens with aromatase‐inducing and 17HSD‐inhibiting activities, licorice can reduce testosterone synthesis and therefore can be used to treat women with PCOS (Kaur et al., 2013). In addition, according to a study on PCOS‐induced mice, licorice extract was shown to improve ovarian morphology, oocyte maturation, and embryonic development in a dose‐dependent manner (100 to 150 mg kg^−1^ day^−1^ for 21 days) compared to the control group (Shamsi et al., 2020).

Licorice extract (3,000 mg kg^−1^ day^−1^ for 6 weeks) has also been reported to reduce endometrial implants in animal models of endometriosis by inhibiting COX‐2 and IL‐6, amplifying the HPG axis, and reducing the expression of vascular endothelial growth factor (VEGF) (Jahromi et al., 2019). However, Räikkönen et al. (2017) declared that prenatal licorice consumption [high (500 mg/week), moderate (250–499 mg/week), and zero–low (0–249 mg/week) doses] by pregnant women increased salivary cortisol levels in newborns in a dose‐dependent manner by inhibiting 11β‐hydroxysteroid dehydrogenase type 2 (the feto‐placental barrier balancing maternal cortisol levels) and simulating the hypothalamic–pituitary–adrenocortical axis; however, it may cause detrimental effects on the development, neuroendocrine function, cognition, pubertal maturation, and psychiatric performance of offspring (Räikkönen et al., 2017). A randomized double‐blinded clinical trial on 60 postmenopausal women showed that licorice extract (10, 20, 50, and 100 mg kg^−1^ day^−1^ for 12 weeks), similar to hormone replacement therapy (0.312 mg conjugated estrogen and 2.5 mg medroxy progesterone daily), reduced the number and severity of hot flashes (i.e., menopausal symptoms) in a dose‐dependent manner compared with the placebo group (Gifford & Reynolds, 2017). A similar randomized double‐blinded controlled trial on 70 postmenopausal women showed that a cream containing 2% licorice extract prevented vaginal atrophy after eight weeks and in addition to reducing vaginal pH, reduced vaginal dryness, soreness, itching, and dyspareunia compared with the placebo group (Sadeghi et al., 2020). Due to the presence of various flavonoids with anticancer properties, the extract of this plant reduces the incidence of endometrial adenocarcinoma by inducing apoptotic pathways and inhibiting inflammatory pathways (i.e., suppressing the cytokines (COX‐2, IL‐1α, and TNF‐α) involved in cancer progression) (Niwa et al., 2007). Therefore, licorice can be used to improve female reproductive function and to treat female reproductive system disorders secondary to the presence of phytoestrogens and flavonoids with beneficial biological effects.

## 4 CONCLUSION

Among the various effective plants in the treatment of various female reproductive disorders, 11 plants with positive effects on female fertility were studied. Due to the presence of various compounds such as polyphenols with many biological activities, these plants are effective in the prevention and treatment of many reproductive disorders such as PCOS, endometriosis, POF, hypothalamic dysfunction, hyperprolactinemia, PID, menopausal symptoms, osteoporosis, and female reproductive related cancers (cervical, ovarian, uterine/endometrial, vaginal and vulvar cancers). After further pharmacological, phytochemical, and toxicological investigations, new and efficacious drugs can be developed by way of comprehensive investigation and of the bioactivity of various compounds purified from extract of this plants.

## CONFLICT OF INTEREST

The authors declare that there is no conflict of interest.

## AUTHOR CONTRIBUTION

**Mohsen Akbaribazm:** Conceptualization (lead); Data curation (lead); Formal analysis (lead); Funding acquisition (lead); Investigation (lead); Methodology (lead); Project administration (lead); Resources (lead); Software (equal); Supervision (equal); Validation (equal); Visualization (equal); Writing‐original draft (lead); Writing‐review & editing (lead). **Nader Goodarzi:** Software (equal); Supervision (equal); Validation (equal); Visualization (equal). **Mohsen Rahimi:** Software (equal); Supervision (equal).

## ETHICAL APPROVAL

The study did not involve any human or animal testing.

## Data Availability

All data generated or analyzed during this study are included in this published article.
